# Enhancing needle puncture detection using high-pass filtering and diffuse reflectance

**DOI:** 10.3389/frobt.2025.1429327

**Published:** 2025-05-06

**Authors:** Rachael L’Orsa, Anupam Bisht, Linhui Yu, Kartikeya Murari, Garnette R. Sutherland, David T. Westwick, Katherine J. Kuchenbecker

**Affiliations:** ^1^ Haptic Intelligence Department, Max Planck Institute for Intelligent Systems, Stuttgart, Germany; ^2^ Department of Electrical and Software Engineering, Schulich School of Engineering, University of Calgary, Calgary, AB, Canada; ^3^ Surgical Performance Laboratory, Project neuroArm, Cumming School of Medicine, University of Calgary, Calgary, AB, Canada; ^4^ Hotchkiss Brain Institute, Cumming School of Medicine, University of Calgary, Calgary, AB, Canada; ^5^ Department of Biomedical Engineering, Schulich School of Engineering, University of Calgary, Calgary, AB, Canada

**Keywords:** tension pneumothorax, needle decompression, data-driven puncture detection, in-bore optical fibers, reflectance, signal processing

## Abstract

**Introduction:**

Chest trauma or disease progression can lead to tension pneumothorax, a condition where mounting pressurization of the pleural cavity (the space between the chest wall and the lungs) leads rapidly to cardiac arrest. In pre-hospital settings, tension pneumothorax is treated by venting the pleural cavity via a needle introduced through the chest wall. Very high failure rates (up to 94.1%) have been reported for pre-hospital needle decompression, however, and the procedure can result in the accidental puncture of critical thoracic tissues because it is performed blind. Instrumented needles could help operators more reliably identify when the tool has entered the target space.

**Methods:**

This paper investigates technical approaches to provide such support; we created an experimental system that acquires needle force and position signals, as well as the diffuse backscattered reflectance from white light carried to and collected from the needle’s tip via two in-bore optical fibers. Data collection occurred while two experimenters inserted a bevel-tipped percutaneous needle into an *ex vivo* porcine rib section simulating human chest anatomy. Four data-driven puncture-detection (DDPD) algorithms from the literature, which are appropriate for use with the variable tool velocities produced by manual insertions, were applied to the resulting data set offline. Grid search was performed across key signal-processing parameters, high-pass filters (HPFs) were applied to examine their impact on puncture detection, and a first exploration of multimodal (ensemble) methods was performed.

**Results:**

Combining high-pass filters with DDPD methods resulted in a 2.7-fold improvement (from 8.2% to 21.9%) in the maximum overall precision (MOP) produced by force signals. Applying this HPF + DDPD scheme to reflectance data streams yielded a peak MOP of 36.4%, and combining reflectance with force generated the best MOP overall (42.1%); these results represent 4.4-fold and 5.1-fold improvements, respectively, over the best MOP produced by the traditional application of DDPD algorithms to force signals alone.

**Discussion:**

These results strongly support the utility of high-pass filters combined with both reflectance-only and multimodal reflectance-plus-force data-driven puncture-detection schemes for needle decompression applications.

## 1 Introduction

**FIGURE 1:**
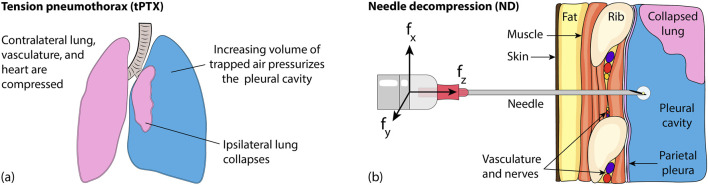
**(a)** Tension pneumothorax interferes with heart motion when pleural cavity pressure is too high. **(b)** Insertion of a hollow bevel-tipped needle between the ribs decompresses the pleural cavity to temporarily alleviate pathological compression.

Tension pneumothorax (tPTX) is an urgent medical condition where pressure from air trapped between a lung and the chest wall cannot be vented (Figure 1a). When the volume of air increases rapidly, it displaces critical structures like the lungs and drastically limits the heart’s ability to pump blood (Spain et al., 2023). Without rapid intervention, tPTX is lethal; it has been identified as a preventable trauma-related cause of death, both for civilians (Kleber et al., 2013) and in combat settings (McPherson et al., 2006).

Needle decompression (ND) can be used in a pre-hospital setting or an emergency department to temporarily vent the trapped volume of air, thus buying time for emergency transport or surgical preparation (Jouneau et al., 2023). To perform ND, the medical operator 1) identifies the recommended needle entry point, 2) angles the needle appropriately, and 3) inserts the needle tip into the trapped volume of air, as illustrated in Figure 1b. The needle bore provides a channel through which the pressurized volume can be vented, but it is neither large enough to release significant quantities of fluids (e.g., blood or pus) if they are present, nor is it secure enough to provide long-term pressure relief (Leatherman et al., 2017). The procedure is considered a *failure* if the tPTX is not immediately diminished, and a *complication* occurs when the needle unintentionally lacerates, punctures, or otherwise damages critical adjacent structures such as the heart, nerves, or key vasculature (Wernick et al., 2015).

Failure rates of up to 94.1% have been reported for pre-hospital ND (Axtman et al., 2019), likely because this time-critical procedure is performed infrequently and without image guidance. Operators must thus rely on the assumption of normal patient anatomy and their sense of touch to estimate when the needle tip has reached its target. Novices are taught to feel for loss-of-resistance (LoR) sensations transmitted through the needle that should indicate the penetration of the tool through the *parietal pleura*, i.e., the membrane that bounds the target volume of air (Figure 1b). Unfortunately, needle-tip forces (and thus LoR sensations) can be obscured by friction forces acting along the needle’s shaft, and friction forces increase with the depth of needle insertion (Washio and Chinzei, 2004). Natural variations in human anatomy (Boyle et al., 2012) and tissue characteristics (Tenorio et al., 2017) further compound the difficulty of needle-puncture detection for operators when they must rely on haptic LoR sensations.

Although puncture detection has not yet been studied for ND, some authors have proposed addressing this challenge for other applications via the use of instrumented needles that explicitly notify the operator when a puncture has occurred (e.g., Gonenc et al., 2014; Gong et al., 2014). We hypothesize that *instrumented needles could likewise assist operators in detecting needle entry into the target space for ND*, thus reducing failure and complication rates, improving patient outcomes, and decreasing the level of difficulty for operators. However, this approach requires puncture-detection algorithms that perform reliably across a range of patient anatomies, tissue characteristics, and the variable-velocity needle-insertion profiles produced during the manual delivery of ND. Therefore, this paper’s contributions are centered on the challenging problem of reliable variable-velocity puncture detection from needle-instrumentation data streams.

A large body of literature exists spanning 30 years of needle-instrumentation research for non-ND applications, e.g., epidurals, venipuncture, and needle steering. However, surprisingly few papers discuss puncture detection directly, and of these, only a small subset present puncture-detection algorithms (see Section 3). Early investigations focused on capturing LoR primarily through force or pressure measurements (e.g., Brett et al., 2000), and they employed force sensors located at the base of the needle due to the difficulty of manufacturing miniaturized, biocompatible, sterilizable sensors that could be placed at the needle tip. However, Washio and Chinzei (2004)’s assertion that needle tip forces are crucial for puncture detection motivated increased research on two sensing modalities that can meet the above-mentioned requirements for sensor collocation: electrical bioimpedance and optical fiber-based methods.

Although there has been a renewed interest in the use of electrical bioimpedance for *needle localization* (i.e., the instrumentation-based confirmation of needle-tip location during insertion via the repeated characterization of tissue properties), the body of literature surrounding this approach is still relatively small. Furthermore, to the best of our knowledge, success with this sensing modality has been demonstrated using only supervised machine-learning methods (Kalvøy et al., 2017; Halonen et al., 2019; Kudashov et al., 2022), and it is unclear whether electrical bioimpedance would be useful in the context of puncture detection when large data sets are not available for model training. In contrast, substantially more literature is available for optical fiber-based methods, and we thus focus on porting these well-developed techniques to the puncture-detection problem.

Since force has traditionally been the sensing modality of choice and is still considered the gold standard for puncture detection, much effort has gone toward producing fiber-based needle-tip force sensors. Abushagur et al. (2014), Su et al. (2017), and Jiang et al. (2023) provide overviews of these developments, yet of the papers reviewed, only those produced by one research group propose novel puncture-detection algorithms. This group’s final publication on the topic (Alamdar et al., 2021) reported mixed success using needle-tip forces for puncture detection, and the group has since moved to image-based puncture-detection algorithms (Kim et al., 2023). Therefore, although needle tip-force sensors can generally provide better puncture features than their non-collocated counterparts, needle-tip force sensing is still not reliable enough for puncture detection in safety-critical applications when used alone, and optical needle-tip force sensors are no exception.

However, in-bore optical fibers also enable the use of other light-based sensing modalities at the needle tip, such as diffuse reflectance spectroscopy (DRS) and optical coherence tomography (OCT). These approaches generally employ one or more fibers to carry light to the needle’s tip and another bundle to collect its reflections. Different tissues absorb and scatter light uniquely based on their optical properties; DRS exploits this behavior by measuring the light scattered by tissue. If its optical properties are characterized in advance, the tissue can be identified from its properties during medical treatment or diagnostics. *In vivo* applications of DRS have often focused on tissue discrimination, either for real-time diagnostics with respect to unhealthy tissues (e.g., Utzinger et al., 2001; Lin et al., 2005; Bensalah et al., 2008; Maryam et al., 2022), or for needle localization during surgical procedures (e.g., Lin et al., 2001; Balthasar et al., 2012, or see Wilson and Eu, 2022 for a recent overview). To the best of our knowledge, only two groups have explicitly examined the use of DRS or OCT for puncture detection, and a third group has proposed DRS for needle localization during ND, as detailed in Section 2.

This paper explores the potential utility of DRS to improve ND with respect to puncture detection. Similar to Gong et al. (2014), our goal is to produce a compact, lightweight, portable adjunct for needle instrumentation that notifies operators to stop advancing the needle when a puncture occurs. The tube thoracostomy literature–which is the closest analog to ND for which extensive research is available–suggests that such an adjunct could be especially useful when operators are inexperienced (Kong et al., 2014; Sritharen et al., 2018), or in cases of unusual patient anatomy (Kerger et al., 2007; Patel et al., 2021).

Specifically, this article carefully examines DRS, signal-processing schemes, four puncture-detection algorithms, and ensemble puncture-detection approaches for their usefulness within the context of ND. Preliminary results from this study were previously reported (L’Orsa et al., 2024), including a basic description of the system, a brief confirmation that low- and high-pass filtering affects the performance of the original DDPD algorithm (Grace, 1995), and initial indications that diffuse reflectance signals could be useful within the context of puncture detection, directly motivating the current study. Here, we substantially expand our analysis by i) optimizing high-pass filter parameters, ii) comparing the performance of an additional three prominent DDPD algorithms, and iii) combining needle-base force and needle-tip reflectance data within multimodal ensemble methods for puncture detection. To the best of our knowledge, this is the first time these three topics have been examined. Section 2 discusses the non-force-based optical-sensing literature relevant to ND, and Section 3 presents the mathematical formulations for the four DDPD algorithms considered herein. Section 4 introduces the experimental apparatus and procedure and then explains how data were processed and analyzed. Section 5 presents results, and Section 6 interprets outcomes, summarizes insights, and suggests future work.

## 2 Optical fiber-based sensing methods for needle decompression

Lee et al. (2021) proposed the use of DRS for needle localization (rather than puncture detection) during ND. Specifically, they described embedding a three-fiber, dual-wavelength, near-infrared spectroscopy system into the bore of an over-the-needle catheter device. Two of the fibers in this system delivered light at dual wavelengths (690 nm and 850 nm) to the needle-tissue interaction site, and the third fiber carried backscattered reflections to a photodiode for detection. The authors reported statistically significant changes in the optical density and the deoxy-hemoglobin/oxy-hemoglobin concentrations estimated for each punctured tissue type. They were able to distinguish the pleural cavity from arteries, veins, muscle, fat, skin, and lung tissues using these data, but the system added a 2-s signal-processing delay. This scale of time delay would either require operators to insert the needle extremely slowly, which would be infeasible given both the patient’s respiratory motion and their urgent need for tPTX treatment, or it would result in significant tool-tip overshoot of the target space. To the best of our knowledge, these authors provide the only description of DRS applied to ND in the literature.

Only two other groups have proposed the use of non-force-based optical sensing modalities for puncture detection in other applications. In one instance, Ting et al. (2010) presented the first in a series of experiments using DRS to identify the target space during epidural insertion. The authors added a bundle of fibers to the bore of an epidural needle and measured the optical spectra during insertion in a porcine model. They identified two wavelengths (532 nm and 650 nm) that differentiated the target space from the tissue bounding it, and they achieved a classification success rate of about 80% using linear discriminant analysis (Lin et al., 2012). The group initially chose to provide operators with puncture notifications based on a pre-determined amplitude threshold (Gong et al., 2014). However, their more recent work simply presents operators with a real-time graphical display of the optical sensor’s output (Lin et al., 2019), which is appropriate only when the needle is inserted slowly.

In contrast, Latus et al. (2021) employed a more sophisticated OCT-based approach to the puncture-detection problem. The relative velocity of tissue at the needle tip was estimated using phase data from OCT A-scans, and an existing puncture-detection algorithm applied to the tissue velocity estimate yielded an overall accuracy between 91% and 94%. However, OCT requires expensive hardware that does not lend itself to portability, as well as complicated signal-interpretation methods that may not be appropriate for pre-hospital deployment. Thus, the literature contains no optical examples relevant to ND, and opportunities remain to explore the use of DRS-based puncture detection in this application.

## 3 Data-driven puncture-detection algorithms

Providing puncture notifications for manual needle insertions requires algorithms that can handle variable-velocity puncture features. Puncture-detection algorithms can be roughly separated into three categories, which we call model-based puncture detection (MBPD), learning-based puncture detection (LBPD), and data-driven puncture detection (DDPD). MBPD approaches assume tool-tissue interaction models or statistical sensor output distributions (e.g., Narayan et al., 2019); they are strongly velocity dependent and are thus inappropriate for manual needle insertions. LBPD approaches apply modern classification techniques (e.g., Kudashov et al., 2022), which require large data sets that are difficult to obtain for ND. DDPD methods do not assume system models or statistical distributions, nor do they require large training data sets. Thus, they are presently the best choice for use with variable-velocity needle insertions.

**FIGURE 2:**
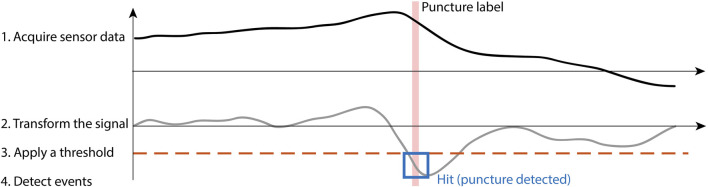
Data-driven puncture detection follows the four-step process of acquiring sensor data, transforming the signal, applying a threshold, and finally detecting events when they occur.

DDPD algorithms involve the four-step process illustrated in Figure 2. These algorithms apply mathematical transformations (e.g., differentiation, multiplication by another signal) to one or more raw data streams; the choice of transform distinguishes between algorithms. A static positive or negative threshold is applied to the transformed output, and puncture events are identified whenever the transformed output exceeds the threshold. These approaches can be formally defined as detecting a puncture when:

$$F\left(s\right)>\lambda_{+}\;or\;F\left(s\right)<\lambda_{-},$$

where s is a set of input signals, F is a mathematical signal transformation, $\lambda_{+}$ is a positive threshold, and $\lambda_{-}$ is a negative threshold. Performance is optimized via threshold selection, and algorithm-specific parameters can also be optimized.

The first accessible introduction of a DDPD approach comes from Grace (1995), which focused on decreasing tool-tip overshoot when a needle punctured a synthetic membrane. Grace’s algorithm transforms its input by estimating the signal’s first time derivative. When applied to force, the algorithm seeks the force-drop features that typically accompany a needle-puncture event, i.e., the LoR feature that clinicians are trained to recognize. We designate this method as “Grace’s approach” moving forward, and it can be mathematically expressed as follows:

$$\frac{df}{dt}<\lambda ,$$ (1)

where f is the force, t is the time, and λ is the threshold (negative here).

Brett et al. (2000) developed a custom filter for their DDPD transform, with the goal of semi-automating epidural needle insertion. The authors used force measurements for robot-driven constant-velocity needle insertions into *ex vivo* porcine spine segments and syringe pressure for handheld variable-velocity needle insertions into human cadavers. We designate their custom filter design as “Brett’s approach”:

ci=∑k=02−1k2kfi−1+k+β∑k=03−1k3kfi−1+kfi⋅maxc>λ, (2)

where c is the filter’s output; i is the discrete sample index; k is an index used for summation, calculation of the binomial coefficients, and discrete sample selection; f is the input signal (e.g., force or pressure); β is an empirically optimized coefficient meant to improve puncture-detection reliability; and λ is the threshold. Note that inclusion of a maximum prevents real-time use.

Gonenc et al. (2016) proposed a series of increasingly complex multimodal puncture-detection algorithms for retinal vein cannulation focused on force and position. However, their system included three separate force sensors located at different points relative to the operator’s hand. Thus, only the portion of their puncture-detection approach described as follows could be employed in our single-force-sensor system:

$$\frac{df}{dt}\cdot \frac{dp}{dt}>\lambda ,$$ (3)

where f is the force, p is the position, t is the time, and λ is the threshold. We refer to this approach as “Gonenc’s approach” henceforth.

Finally, Kowal (2017) presented a novel DDPD algorithm for the detection of micro-needle punctures into murine skin and lymphatic tissue. The algorithm loads consecutive discrete sensor samples (e.g., force) into a ring buffer of even length, and it transforms the input signal by calculating the absolute difference between the averages of the first and last halves of the buffer contents. We designate this method as “Kowal’s approach”:

$$\Delta F_{\text{avg}}=\frac{2}{N}\left|\overset{N/2}{\underset{i=1}{\sum }}B_{i}\;-\overset{N}{\underset{j=N/2+1}{\sum }}B_{j}\right|>\lambda ,$$ (4)

where N is the buffer length, i and j are summation indices, λ is the threshold, and $B_{i}$ and $B_{j}$ are the older and newer buffer contents, respectively.

## 4 Materials and methods

### 4.1 Experimental apparatus

**FIGURE 3:**
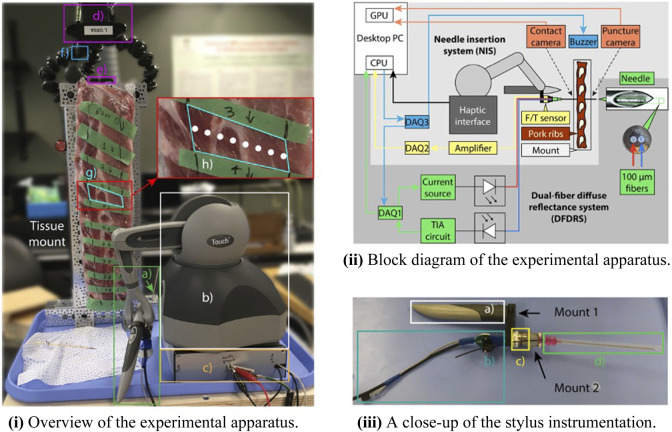
**(i)** The experimental apparatus consists of **a)** an instrumented needle, **b)** a haptic interface, **c)** a Faraday cage, **d)** a contact camera, **e)** a puncture camera, and **f)** a buzzer. The needle interacts with a row of **g)** intercostal tissue, and the operator attempts to space insertions as illustrated by the white dots in **h)**. **(ii)** Block diagram of the experimental apparatus. **(iii)** The haptic interface's **j)** stylus suspends **k)** a custom ferrule holder, the LED's supply PCB, and **l)** the force/torque sensor, via custom mount 1. The second custom mount attaches the force/torque sensor to **m)** the needle and provides an angled channel through which the fibers exit the base of the needle hub.

The experimental apparatus contained two subsystems: a dual-fiber diffuse reflectance system (DFDRS) that produces a reflectance data stream and a needle-insertion system (NIS) that collects all other data streams. The experimental apparatus and its block diagram can be found in Figures 3i, ii, respectively.

#### 4.1.1 Dual-fiber diffuse reflectance system

The DFDRS transmits light to the needle’s tip with one optical fiber and collects that light’s diffuse reflections with another. The fibers were both approximately 45 cm long with 100 μ m cores (UM22-100 from Thorlabs, United States), and they were secured in a 26G stainless steel tube such that the source-detector separation (SDS) was about 125 μ m. The steel tube was positioned inside the bore of a 9 cm 18G percutaneous access needle (Cook Medical, United States). The exposed fiber ends emerged at the needle tip, and the tube was cemented in place using epoxy. Both ends of both fibers were cleaved and polished with a fiber polisher (Krell Technologies, United States) prior to insertion into the metal tube, and the free ends emerging from the needle’s hub were terminated with 2.5 mm ferrules (Thorlabs, United States).

A warm white LED producing wavelengths within an envelope of about 410–780 nm (XLamp XQ-E from CreeLED, United States) was butt-coupled to the delivery fiber using custom components. The LED was driven with a Howland current source generating about 40 mA of peak-to-peak current around a 20 mA average. This design led to about 200 μ W optical power incident on the tissue, which is sufficient for reflectance measurements (Bisht et al., 2024). A homodyne measurement scheme was applied to minimize the effects of ambient light, and the sinusoidal modulation frequency of the drive current was set to 143 Hz. The collection fiber was butt-coupled to a surface-mount photodiode (VBPW34S from Vishay Semiconductor Opto Division, United States) mounted on a PCB with a transimpedance amplifier (TIA) of 1 M Ω gain and 1 kHz bandwidth. A multifunction I/O device (DAQ1: USB-6361 from National Instruments, United States) supplied the Howland circuit’s input voltage and sampled the output of the TIA circuit at a rate of 2860 Hz. Supplementary Appendix 1.1 characterizes optical aspects of the DFDRS.

#### 4.1.2 Needle-insertion system

A desktop haptic interface (Touch from 3D Systems, United States) formed the base of this subsystem and transmitted its joint encoder values to the computer directly via USB. The device’s motors were not powered during this study. The haptic interface sat (unfixed) on top of the DFDRS’s Faraday cage, which in turn sat (also unfixed) atop wooden risers. The risers could be reconfigured to alter the relative height of the haptic interface, which was necessary to align the haptic interface’s workspace with each row of intercostal tissue in the *ex vivo* sample, but it precluded precise registration between the two.

A custom mount suspended a 6-DoF force/torque sensor (Nano17 Titanium from ATI Industrial Automation, United States) below the haptic interface’s stylus (Figure 3iii). A second custom mount secured the base of the needle to the force/torque sensor’s tool adapter plate at its sensing reference frame origin. The computer collected the amplified force/torque measurements via a second multifunction I/O device (DAQ2: PCI-6280 from National Instruments, United States).

Commercially available *ex vivo* baby back pork ribs served as the tissue sample, as recommended by Joseph and Verelst (2021). The ribs were secured vertically in the tissue mount with their pleural membrane facing away from the haptic interface. Two USB webcams (C922 Pro HD Stream from Logitech, Switzerland) provided the image streams for the apparatus, and a piezo buzzer was fastened between the two cameras. A signal from a third multifunction I/O device (DAQ3: Q8-USB from Quanser, Canada) commanded the buzzer to produce sounds that were used to synchronize the image and data stream collection. DAQ3 also enabled synchronization of the DFDRS and NIS data streams by feeding a clock signal to DAQ1.

#### 4.1.3 Data collection software and computer

Free, open-source video-streaming software (OBS Studio from H. Bailey and OBS Project contributors) recorded the image streams from the two cameras side-by-side in the same canvas at a rate of 60 frames per second (fps). All NIS data streams were collected using real-time control software (QUARC 2019 SP1 from Quanser, Canada, embedded in Simulink 10.0 from MathWorks, United States), which produced an average sampling rate of 1,000.3 ± 0.1 Hz. However, DAQ1 was not compatible with this software, so custom C code was used to save the data it recorded. A custom-built desktop Windows 10 computer with an i9-9900 processor, 64 GB of RAM, and an NVIDIA GeForce RTX 2060 SUPER graphics card recorded all image and data streams.

### 4.2 Procedure

On the day of the experiment, the refrigerated tissue sample was procured, warmed naturally to room temperature (21 ° C), and secured in the tissue mount. Two authors served as operators, and they practiced the puncture task on the first row of intercostal tissue. For each of the remaining nine rows of tissue, the following occurred:

1. A MATLAB script randomly assigned the operator role.
2. The operator confirmed that the haptic interface’s workspace could reach the appropriate row of tissue.
3. The cameras were manually centered and focused in the image-streaming software.
4. Image streaming and data collection were initiated.
5. Five seconds of sensor outputs were collected, and the mean of each data stream was subtracted to remove sensor bias.
6. Two synchronization buzzes were issued.
7. The operator inserted the needle as many times as possible from left to right in their row of intercostal tissue.

Operators were instructed to i) keep a horizontal distance of about 1 cm between neighboring insertions, ii) maintain a perpendicular alignment between the needle and the tissue sample, iii) maintain a parallel alignment between the needle and the floor, iv) insert the needle at the vertical midpoint between the two ribs bounding the row of intercostal tissues, and v) halt needle advancement when they feel a ‘pop’. This last instruction is the primary guidance given to medical professionals when they learn ND, and it is meant to help an operator identify when they’ve punctured the parietal pleura.

The webcam images were not visible to the operator, no form of cleaning was applied to the needle between insertions, and the tissue sample’s parietal pleura was misted frequently with water to maintain its moisture levels. This procedure yielded recordings of 93 potential needle-puncture trials.

### 4.3 Data processing

The analyses in this paper consider only the reflectance (R) , the axial insertion force (f) , and the Cartesian positions ( $p_{x}$ , $p_{y}$ , and $p_{z}$ ) collected from the haptic interface. For every trial, the synchronization buzzes were manually identified in the first time derivative of the audio signal and aligned with the matching buzzer activation voltage command from DAQ3. Each insertion was segmented, and the image streams were manually labeled by an experimenter. All data streams and labels are publicly available (L’Orsa et al., 2025).

#### 4.3.1 Event labeling

Single-frame labels were identified for each of five events, which we abbreviate as events $E_{1}$ through $E_{5}$ :

1. **Contact** $\left(E_{1}\right)$ : First frame where the needle appears to contact the tissue (as seen in the contact camera).
2. **Tip puncture** $\left(E_{2}\right)$ : First frame where the needle tip appears to puncture through the parietal pleura (as seen in the puncture camera).
3. **Bevel puncture** $\left(E_{3}\right)$ : First frame where the entire needle bevel is visible from the puncture camera.
4. **Shaft puncture** $\left(E_{4}\right)$ : First frame where the needle shaft (below the bevel) is visible from the puncture camera.
5. **Maximum advancement** $\left(E_{5}\right)$ : Last frame where the needle visibly advances.

Tip puncture frame labels are used as ground-truth puncture events in this work.

#### 4.3.2 Insertion phases and metrics

An insertion includes all data samples from the last sample before $E_{1}$ to the first sample after $E_{5}$ , a puncture includes all data samples from the last sample before $E_{2}$ to the first sample after $E_{4}$ , and an operator’s reaction period includes all data samples from the last sample before $E_{4}$ to the first sample after $E_{5}$ . Therefore, the duration $\left(t_{i}\right)$ of an insertion, the duration of a puncture $\left(t_{p}\right)$ , and the time taken for an operator to react to a puncture before stopping the needle (i.e., their reaction time, $t_{r}$ ) were calculated as the difference between the time stamps of the appropriate first and last samples.

The needle’s displacement along its axis of insertion was approximated by zeroing the Cartesian position vectors at the start of each insertion and then calculating the magnitude of the vector (‖p‖) . The needle penetration depth was approximated as $p_{d}=\|p{\|}_{e_{5}}-\|p{\|}_{e_{1}}$ , and the tool-tip overshoot was estimated as OS $=\|p{\|}_{e_{5}}-\|p{\|}_{e_{2}}$ . The needle velocity was estimated as $v=\frac{d\|p\|}{dt}$ , where a positive value indicates advancement. A moving-average low-pass filter (LPF) with window length $l_{\text{win}}=219$ was applied to v prior to statistical analysis.

#### 4.3.3 Reflectance demodulation

Reflectance data were demodulated by first calculating the in-phase component $\left(R_{\cos}=\cos\left(2\pi f_{i}\cdot t_{R}\right)\cdot R\right)$ of the reflectance and its quadrature counterpart $\left(R_{\sin}=\sin\left(2\pi f_{i}\cdot t_{R}\right)\cdot R\right)$ , where $f_{i}=$ 143 Hz is the frequency of the LED’s drive current and $t_{R}$ is the reflectance sample time. A Butterworth LPF with a cutoff frequency of $f_{c}$ = 6 Hz isolated the DC components of $R_{\cos}$ and $R_{\sin}$ , yielding $R_{\cos}^{\prime }$ and $R_{\sin}^{\prime }$ . Finally, the demodulated reflectance was calculated as $R_{\text{demod}}=\sqrt{{\left(R_{\sin}^{\prime }\right)}^{2}+{\left(R_{\cos}^{\prime }\right)}^{2}}$ .

Partial and full demodulation was accomplished using first- and sixth-order Butterworth LPFs, respectively, in the demodulation process described above. We also calculated the period-wise average of the extrema (ae) of the raw and partially demodulated reflectance signal. To do so, the signal minimum and maximum values were found within sliding windows of 30 reflectance samples, and the average within each window was calculated as $R_{\text{ae,win}}=\left(R_{\text{min,win}}+R_{\text{max,win}}\right)/2$ .

We designate the raw $\left(R_{1}\right)$ , partially demodulated $\left(R_{2}\right)$ , fully demodulated $\left(R_{3}\right)$ , sliding-windowed ae of the raw $\left(R_{4}\right)$ , and sliding-windowed ae of the partially demodulated $\left(R_{5}\right)$ reflectance signals via subscripts one through five, respectively. We refer to the set $\left\{R_{1},\;R_{2},\;R_{3},\;R_{4},\;R_{5}\right\}$ as R , and individual elements are called signal *variations*.

#### 4.3.4 Low-pass filtering

Two different LPFs were applied to both reflectance and force signals: a moving average filter and a fifth-order Savitzky-Golay filter. We designate signals filtered with the moving average and Savitzky-Golay filters via m and s subscripts, respectively, and we indicate the $l_{\text{win}}$ used to calculate the output of a filter by adding it to the subscript. For example, $p_{x,m9}$ represents the x -axis position, low-pass filtered using a moving average filter with a window length of nine samples. Note that unfiltered force data streams are indicated via ‘raw’ in the subscript, while we use ‘u’ to indicate elements of R that have not been explicitly low-pass filtered (LPF’ed), since $R_{2}$ through $R_{5}$ have been modified compared to the raw values of $R_{1}$ .

We applied a grid search to optimize DDPD performance using $l_{\text{win}}=$ {9, 16.7, 25, 50, 75, 220} ms, which translated into {9, 17, 25, 49, 75, 219} samples for force and {25, 47, 71, 143, 215, 629} samples for reflectance. Thus, for example, the full set of partially demodulated reflectance signals after low-pass filtering (LPF’ing) is represented as follows:

$$\begin{array}{l} R_{2,\text{LPF}}=\left\{\begin{array}{cccccccc} R_{2,u}, & R_{2,m25}, & R_{2,m47}, & R_{2,m71}, & R_{2,m143}, & R_{2,m215}, & R_{2,m629}, & \ldots \end{array}\right.\\ \left.\begin{array}{cccccc} \;R_{2,s25}, & R_{2,s47}, & R_{2,s71}, & R_{2,s143}, & R_{2,s215}, & R_{2,s629} \end{array}\right\} \end{array}$$

We refer to a set of LPF’ed signals as L , and each element in L is also called a signal variation.

#### 4.3.5 DDPD signal transformations

DDPD outputs were calculated for the raw force signal or each signal in R or L , as appropriate, as described below.

##### 4.3.5.1 Grace’s approach

The first time derivatives in Equation 1 were approximated via single backwards differencing. A Hampel filter with three neighbors was applied to force derivatives to reduce the effects of sampling rate variability, after which L was calculated. The “der” subscript is used henceforth to describe signals to which Grace’s approach has been applied.

##### 4.3.5.2 Brett’s approach

The original parameters listed by Brett et al. (2000) were used for Brett’s approach, i.e., β=0.01 and binomial coefficients $\left(\begin{array}{c} 2\\ k \end{array}\right)$ and $\left(\begin{array}{c} 3\\ k \end{array}\right)$ for the first and second summations, respectively, in Equation 2. The “bre” subscript is used henceforth to describe signals to which Brett’s approach has been applied. Rather than L , raw signals served as the input to Brett’s approach.

##### 4.3.5.3 Gonenc’s approach

As shown in Equation 3, Gonenc’s approach thresholds the product of two input signals: $\frac{df}{dt}$ and $\frac{dp}{dt}$ . Since the haptic interface was not registered to the tissue sample, three variations of Gonenc’s approach were calculated when either force or reflectance served as the first algorithm input. The “gon1,” “gon2,” and “gon3” subscripts are used henceforth to identify the use of $p_{x}$ , $p_{y}$ , and $p_{z}$ , respectively, as the second algorithm input.

When Gonenc’s approach was applied to reflectance signals, another four variations (“gon4” through “gon7”) were included to examine how crucial force signals are for the success of Gonenc’s approach. Table 1 clarifies these input signal combinations and their identifiers.

**TABLE 1:** Input signal combinations defining the various versions of Gonenc’s method explored. Column shading is used to differentiate Gonenc’s original approach (as applied using Cartesian position components) from the new variations we explored.

	gon1	gon2	gon3	gon4	gon5	gon6	gon7
Signal 1	$\frac{df}{dt}$ or $\frac{dR}{dt}$	$\frac{df}{dt}$ or $\frac{dR}{dt}$	$\frac{df}{dt}$ or $\frac{dR}{dt}$	$\frac{dR}{dt}$	$\frac{dR}{dt}$	$\frac{dR}{dt}$	$\frac{dR}{dt}$
Signal 2	$\frac{dp_{x}}{dt}$	$\frac{dp_{y}}{dt}$	$\frac{dp_{z}}{dt}$	$\frac{df}{dt}$	$\left(\frac{df}{dt}\cdot \frac{dp_{x}}{dt}\right)$	$\left(\frac{df}{dt}\cdot \frac{dp_{y}}{dt}\right)$	$\left(\frac{df}{dt}\cdot \frac{dp_{z}}{dt}\right)$

Regardless of the choice of first input, the second input to the algorithm was always LPF’ed using a moving average filter with $l_{\text{win}}=219$ to minimize the amplification of signal noise. Since the reflectance and force/position sampling rates differed, all input signals were decimated by finding their maximum signal envelopes within a given video frame, such that frame-wise products could be calculated within the algorithm itself.

##### 4.3.5.4 Kowal’s approach

Applying Equation 4 requires judicious selection of the buffer size. A buffer size of N=40 samples was selected for modulated reflectance signals because this number represents roughly two modulation periods given the 143 Hz drive frequency. Buffer sizes of N=20 samples and N=6 samples were selected for demodulated reflectance signals and force signals, respectively. The “kow” subscript is used henceforth to describe signals to which Kowal’s approach has been applied.

#### 4.3.6 High-pass filtering

Finally, a set of high-pass filters (HPFs) was applied to the output of each DDPD algorithm to investigate the recommendation of seeking high-frequency post-puncture needle/tissue oscillations (Kowal, 2017). Preliminary coarse grid searches in the training data set indicated that cutoff frequencies $f_{c}$ of around 200 Hz produced the best DDPD results (L’Orsa et al., 2024), so we explored $f_{c}=\left\{\begin{array}{ccc} 175, & 200, & 225 \end{array}\right\}$ Hz in this work. However, these cutoff frequencies exceed the Nyquist frequency when Gonenc’s approach is applied to reflectance signals, so $f_{c}=\left\{\begin{array}{ccc} 5, & 15, & 25 \end{array}\right\}$ Hz were used in those groups. We indicate the application of HPFs using the subscript h coupled with the appropriate $f_{c}$ . For example, $f_{\text{raw}-\text{gon}2-h200}$ denotes the raw needle insertion force, multiplied by the y -axis position, and subsequently high-pass filtered (HPF’ed) with $f_{c}=200$ Hz. We refer to a set of HPF’ed signals as H , and as with R and L , each element in H is also called a signal variation.

### 4.4 Algorithm application

After the removal of invalid insertions (Supplementary Appendix 1.2), a 50/20/30 split was applied randomly to the remaining 81 insertions on a per-row basis to produce training/validation/test sets of insertions with equal row representation.

#### 4.4.1 Threshold sets

For each DDPD method, we searched a grid of 1,000 positive and 1,000 negative thresholds to identify one optimal threshold, $\lambda_{\text{MOP}}^{*}$ , for each DDPD method (see Section 4.4.3). To generate a set of thresholds for a given signal variation, the appropriate global signal extremum (the global maximum, $M_{\max}$ , for positive threshold sets and the global minimum, $M_{\min}$ , for negative threshold sets) was found in the training set of insertions. A negative or positive threshold increment $\left(\delta_{\lambda }\right)$ was calculated that would produce 999 thresholds spanning from 0% to 99.999% of the global signal extremum, and then a final threshold increment was added to address the scenario where a puncture cannot be detected. Therefore, negative threshold sets spanned $\lambda_{-}=\left\{x\in R\;:\;0\leq x\leq \left(M_{\min}+\delta_{\lambda -}\right)\;and\;\frac{x}{\delta_{\lambda -}}\in Z\right\}$ and positive threshold sets spanned $\lambda_{+}=\{x\in R\;:\;0\leq x\leq \left(M_{\max}+\delta_{\lambda +}\right)\;and\;\frac{x}{\delta_{\lambda +}}\in Z\}$ , and the full set of thresholds describing the optimization search space is $\lambda =\left\{\lambda_{-},\;\lambda_{+}\right\}$ .

#### 4.4.2 Event windows

The needle becomes visible beyond the parietal pleura only in the frame that follows puncture occurrence. Therefore, the actual puncture event happens before the $E_{2}$ ground-truth frame label, so we define an *event window* as a block of data samples that precedes $E_{2}$ and is most likely to contain the true puncture event. We set the length of an event window to three video frames (recorded at 60 fps) because the streaming software generally alternates between cameras but drops a frame at least once per second. This behavior can intermittently result in a scenario where a new frame is not obtained from the puncture camera until two frame periods have passed.

**FIGURE 4:**
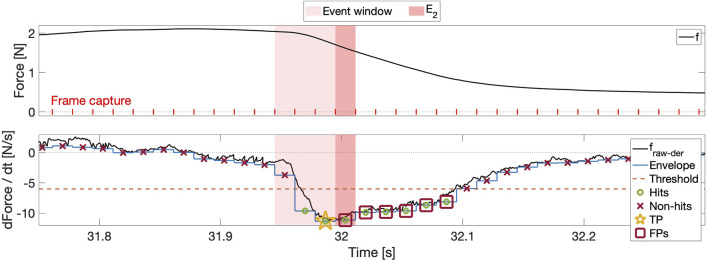
Top: The force generated during insertion 3 in row 2, with vertical ticks denoting video frames and background coloration indicating both tip puncture $\left(E_{2}\right)$ and the event window. Bottom: $\frac{df}{dt}$ , shown with its negative frame-wise signal envelope and an arbitrary negative threshold, generates algorithm hits when the envelope exceeds the threshold in the negative direction. The most prominent hit in the event window is designated as the true positive (TP), and hits outside the event window are deemed false positives (FPs).

Ground-truth labels are frames rather than samples, which necessitates the calculation of the appropriate (negative or positive) frame-wise envelope for any given signal variation prior to threshold application. An algorithm *hit* occurs in each frame where the envelope exceeds the threshold in the appropriate (negative or positive) direction, and the algorithm achieves a TP when a hit occurs within the event window. To avoid generating more than one TP per insertion due to the three-frame window length, TP status is assigned to the frame in which the envelope exceeds the threshold by the largest amount, and any other hit-containing frames in the event window are designated as true negatives (TNs). The outcome of this classification process is illustrated in the bottom subplot in Figure 4, where TNs are omitted to improve visibility. If the threshold exceeded the TP, then this frame would be designated as the unique false negative (FN).

#### 4.4.3 Performance metric

Precision $\left(\frac{\text{TP}}{\text{TP}+\text{FP}}\right)$ is zero (0%) when the lone positively labeled frame is missed and one (100%) when it is found without any FPs. If both the TP and a single FP are identified, the precision drops to 0.5 (50%), and it approaches zero as the number of FPs increases. Therefore, this metric is stringent; higher precision is desirable, and a precision of one (i.e., 100%) is the goal. The precision was calculated at each threshold in λ for each insertion, and the threshold-specific precision was averaged across all insertions in a given set of insertions. This process yields 2000 threshold-averaged precision (TAP) values for λ , multiplied by 13 LPF variations, four HPF variations, and six signals ( f or R ).

**FIGURE 5:**
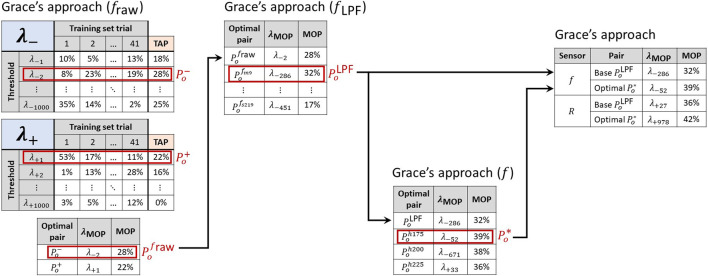
A sequential process identifies the most performant signal-threshold pairs $\left(P_{o}\right)$ in the training set. First, the threshold-averaged precision (TAP) is calculated for each threshold. The largest TAP value is the maximum overall precision (MOP) for each of $\lambda_{-}$ and $\lambda_{+}$ , and it confirms which threshold sign performs best for a given signal variation. The $P_{o}$ with the largest MOP in L yields the base pair for a given sensor. The largest MOP across L and H indicates a sensor’s most performant signal-threshold pair overall $\left(P_{o}^{*}\right)$ , and these $P_{o}^{*}$ can then be compared between sensing modalities. The values listed in this figure are for illustrative purposes only and do not reflect actual results.

We call the maximum value of the TAP for $\lambda_{-}$ or $\lambda_{+}$ the *maximum overall precision* (MOP), and we designate the threshold at which it is found with the MOP subscript $\left(\lambda_{\text{MOP}}\right)$ . A signal variation and its $\lambda_{\text{MOP}}$ are an *optimal pair* $\left(P_{o}\right)$ . Thus, $P_{o}^{-}$ and $P_{o}^{+}$ identify the $P_{o}$ that yield the largest MOP in $\lambda_{-}$ and $\lambda_{+}$ , respectively. The most performant $P_{o}$ in L is called $P_{o}^{\text{LPF}}$ . Similarly, the most performant $P_{o}$ in H are denoted using the appropriate HPF identifiers, e.g., $P_{o}^{\text{h}200}$ . Finally, $P_{o}*$ indicates the most performant $P_{o}$ overall for a given DDPD approach, i.e., $P_{o}^{*}=\max\left(\begin{array}{cccc} P_{o}^{\text{LPF}} & P_{o}^{\mathrm{h175}} & P_{o}^{\mathrm{h200}} & P_{o}^{\mathrm{h225}} \end{array}\right)$ . Figure 5 clarifies how each of these identifiers is assigned during the analysis of a single DDPD approach. Note that $P_{o}^{\text{LPF}}$ represent the best performance achieved by the original DDPD algorithms, and thus they provide the baseline puncture-detection performance. In this capacity, we refer to them as *base pairs*, and we use them to assess the performance effects of HPFs and ensembles.

#### 4.4.4 Ensemble selection criteria

The most performant $P_{o}*$ from the training set were identified according to the procedure outlined in Supplementary Appendix 1.3. These $P_{o}*$ were evaluated in the validation set of insertions, and the degree of generalization for each $P_{o}*$ was estimated based on the percent change in MOP $\left(\Delta_{\text{MOP}}\right)$ between the training and validation sets. Any $P_{o}*$ with MOP <10% in either the training or validation set was discarded, as was any $P_{o}$ with $\Delta_{\text{MOP}}<-10\%$ . Next, the average of the MOP* values obtained in training and validation was used as a decision-making metric (DM), and $P_{o}*$ with DM <35 were also discarded. These limits were selected arbitrarily as a means of decreasing the number of $P_{o}*$ for advancement. Finally, the remaining subset of $P_{o}*$ was ranked in descending order according to DM. The top-ranked force $P_{o}*$ , the top-ranked reflectance $P_{o}*$ , and the next-highest-ranked $P_{o}*$ overall (across all DDPD methods) were selected from this subset for ensemble testing.

#### 4.4.5 Ensembles

Four types of basic signal combinations were considered, where each combination takes as input the frame-wise envelopes of two or more signal variations from the three $P_{o}*$ identified via the ensemble selection criteria:

1. **Multiplication** (E_M_): the input envelopes are multiplied together, element by element (as in Gonenc’s original approach, but without the focus on force and position as input signals).
2. **Weighted sum** (E_WS_): a weight is chosen for each input envelope such that they are all transformed to a similar order of magnitude, and then the weighted envelopes are summed together in an element-wise fashion.
3. **Logical operations** (E_LO_): AND, OR, and XOR operations are applied to the hit/no-hit arrays generated by applying $\lambda_{\text{MOP}}$ to each input envelope.
4. **Voting** (E_SUM_): the individual hit/no-hit arrays generated by $\lambda_{\text{MOP}}$ application are summed on an element-wise basis, and a majority vote is applied to this sum.

We compared all possible combinations of the three ensemble input signals, where the global extrema for the E_M_ and E_WS_ signals were found for threshold set construction as described in Section 4.4.1. The ${\lambda *}_{\text{MOP}}$ was selected from the training set for E_M_ and E_WS_, and the ${\lambda *}_{\text{MOP}}$ for individual ensemble input signals were used for E_LO_ and E_SUM_. Ensemble results were collected for both the training and validation sets, and the relevant portions of the selection criteria described in Supplementary Appendix 1.3 were applied. Finally, the most performant base pairs, the most performant optimal pairs overall, and all ensemble results were compared in the test set of insertions.

## 5 Results

### 5.1 Descriptive statistics

Basic descriptive statistics are provided for f and v on a per-operator basis in Table 2. The Shapiro-Wilk test and Q-Q plots suggested non-normal distributions for the mean and maximum f and v , so the Kruskal–Wallis test was used to confirm whether these values differed between operators with a level of significance of α=0.05 . The test confirmed that the two operators produced similar mean forces (p=0.58) but different maximum forces (p=0.013) , mean velocities (p=0.007) , and maximum velocities (p=0.00065) over the course of their insertions, i.e., from $E_{1}$ to $E_{5}$ .

**TABLE 2:** Top: The number of rows punctured $\left(n_{r}\right)$ , the total number of insertions across all rows $\left(n_{i}\right)$ , and the mean and maximum force (f) and velocity (v) during insertion for each operator. Bottom: The mean and maximum force (f) and velocity (v) during puncture for each operator. Standard deviations are included as appropriate.

Insertion						
Operator	$n_{r}$	$n_{i}$	$\bar{f}$ [N]	max(f) [N] *	$\bar{v}$ [mm/s]**	max(v) [mm/s]***
1	5	52	0.74 ± 0.13	1.50 ± 0.27	6.12 ± 2.47	14.12 ± 6.91
2	4	29	0.77 ± 0.16	1.42 ± 0.43	8.17 ± 3.21	18.28 ± 6.90
Both	9	81	0.75 ± 0.14	1.49 ± 0.38	6.85 ± 2.89	16.10 ± 8.39

Puncture				
Operator	$\bar{f}$ [N]***	max(f) [N]	$\bar{v}$ [mm/s]**	max(v) [mm/s]*
1	0.75 ± 0.13	1.30 ± 0.21	8.20 ± 2.90	12.20 ± 3.98
2	0.98 ± 0.16	1.37 ± 0.45	11.54 ± 5.84	15.11 ± 6.95
Both	0.83 ± 0.14	1.35 ± 0.36	9.40 ± 4.43	13.59 ± 6.18

The annotations *, **, and *** denote statistically significant differences between operators with p < 0.05, 0.01, and 0.001, respectively.

Table 2 also contains the mean and maximum values of f and v during puncture, i.e., from $E_{2}$ to $E_{4}$ . The Kruskal–Wallis test showed statistically significant differences between the mean puncture forces, mean puncture velocities, and maximum puncture velocities produced by operators ( p<0.0001 , p=0.0014 , and p=0.046 , respectively), but not their maximum puncture forces (p=0.84) .

Table 3 shows performance metrics averaged across all insertions for each operator. The tool-tip overshoot is broken out into its extrema for emphasis, and the maximum overshoot is highlighted because it represents the source of complications in ND. The Kruskal–Wallis test suggested that the average $p_{d}$ and OS produced by the two operators were not significantly different ( p=0.82 and p=0.22 , respectively), but their average $t_{i}$ , $t_{p}$ , and $t_{r}$ were ( p=0.0014 , p=0.00073 , and p<0.0001 , respectively). Note that it is possible for operators to produce very small tool-tip overshoots (i.e., less than 1 mm) under ideal conditions, but the maximum values produced herein (over 1 cm, on average) could be dangerous for an ND recipient when the needle is incorrectly positioned or angled.

**TABLE 3:** The penetration depth $\left(p_{d}\right)$ ; minimum, mean, and maximum tool-tip overshoot (OS); insertion duration $\left(t_{i}\right)$ ; puncture duration $\left(t_{p}\right)$ ; and reaction time $\left(t_{r}\right)$ of each operator, averaged across all needle insertions. Standard deviations are included for all variables except OS.

Operator	$p_{d}$ [mm]	min(OS) [mm]	$\bar{\text{OS}}$ [mm]	max(OS) [mm]	$t_{i}$ [s]**	$t_{p}$ [s]***	$t_{r}$ [s]***
1	15.58 ± 3.59	1.07	3.71	12.80	2.76 ± 0.94	0.13 ± 0.06	0.41 ± 0.28
2	15.29 ± 2.08	0.61	2.91	8.74	2.03 ± 0.89	0.09 ± 0.04	0.17 ± 0.05
Both	15.48 ± 3.10	0.61	3.42	12.80	2.50 ± 0.98	0.11 ± 0.06	0.32 ± 0.26

### 5.2 Training set results

Since only the most performant optimal pairs were advanced to the test set of insertions, performance comparisons between algorithms and factor effects were evaluated in the training set of insertions.

#### 5.2.1 Comparison of algorithms

**FIGURE 6:**
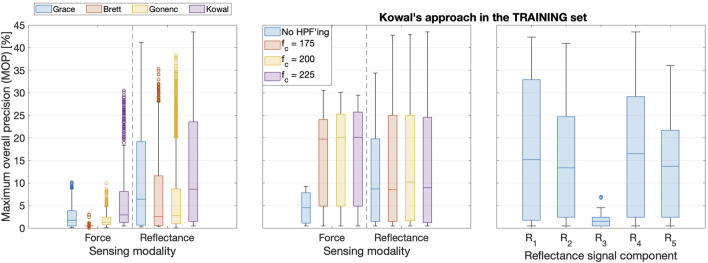
Left: Boxplots comparing training set results for all four DDPD algorithms. Boxes span the interquartile range (IQR: from the 25th to the 75th percentiles), with a bar indicating the median. Whiskers span the range up to 1.5 ⋅ IQR, and outliers appear as circles. The vast majority of signal-threshold pairs perform poorly, and the top outliers represent the most performant optimal pairs. Middle: HPF’ing signals prior to puncture detection produces substantial performance improvements, though the cut-off frequency $\left(f_{c}\right)$ should be tuned for each signal. Right: $R_{4}$ is the most performant reflectance signal, whereas $R_{3}$ retains substantially fewer puncture features.

The left subplot in Figure 6 shows a box plot containing all signal-threshold pairs in the training set of insertions. The results are grouped according to the sensor and the DDPD approach, which highlights that *Kowal’s approach performed the best in both force and reflectance data streams*.

#### 5.2.2 High-pass filter effects

The middle subplot in Figure 6 clarifies that *HPFs improve performance* when the most performant DDPD algorithm (Kowal’s approach) is applied to data from either sensor. This subplot also shows that increasing $f_{c}$ decreases the MOP for force but slightly increases it for reflectance; hence, HPFs must be tuned separately for each sensing modality. These training set results with respect to high-pass filtering (HPF’ing) are representative of all four DDPD methods.

#### 5.2.3 Comparison of reflectance signal variations

**FIGURE 7:**
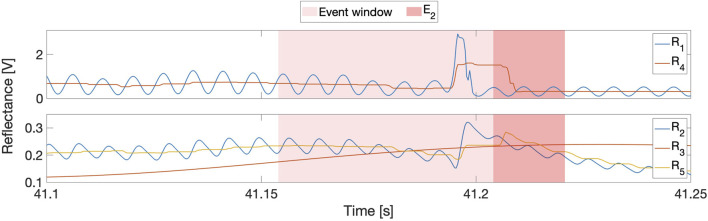
Top: Comparison of reflectance signal variations at tip puncture $\left(E_{2}\right)$ during insertion 5 of row 2. $R_{1}$ is the raw signal, and $R_{4}$ is the sliding-windowed average of its extrema. Bottom: $R_{2}$ and $R_{3}$ are the partially and fully demodulated signals, and $R_{5}$ is the sliding-windowed average of the extrema of $R_{2}$ .

The top subplot in Figure 7 shows how the modulated signal $\left(R_{1}\right)$ and its sliding-window-extrema-averaged counterpart $\left(R_{4}\right)$ changed at tip puncture during a representative insertion, whereas the bottom subplot shows the response of the demodulated signals. As expected, both $R_{1}$ and $R_{2}$ contain substantial sinusoidal content. $R_{1}$ contains a prominent puncture feature that is aligned with the event window; $R_{4}$ is much smoother than its raw counterpart, but the sliding-window averaging both attenuates the puncture feature and smears it into the next frame. A similar pattern emerges for $R_{2}$ and $R_{5}$ , though the smoothing applied to $R_{2}$ during partial demodulation further delays, attenuates, and widens the puncture feature. Indeed, these repeated smoothing operations delay $R_{5}$ ’s small puncture feature so much that it is no longer aligned with the event window. Therefore, *although smoothing is desirable to improve signal-to-noise ratios, it can delay, decrease, and even destroy puncture features* and must thus be applied very carefully within DDPD schemes. A prime example of this effect can be seen in the fully demodulated $R_{3}$ data stream, which does not contain a puncture feature at all. Unsurprisingly, $R_{3}$ performs very poorly compared to other R signal variations, as shown in the right subplot of Figure 6.

#### 5.2.4 Gonenc’s method in reflectance

**FIGURE 8:**
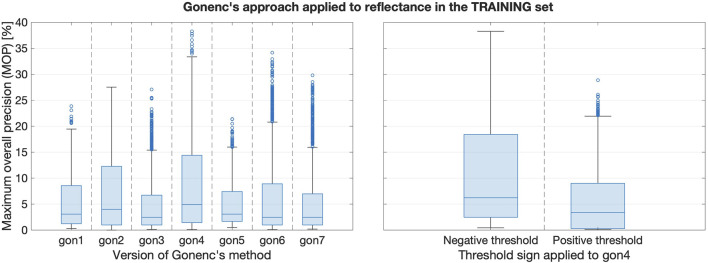
Left: Boxplots comparing training set results when reflectance was the first input to Gonenc’s algorithm. Right: The gon4 version with negative thresholds performs the best.

Recall that it was unclear how best to apply Gonenc’s method to reflectance data streams. Figure 8’s left subplot compares the performance of the seven reflectance-based Gonenc variations (see Table 1). Clearly, $\frac{dR}{dt}\cdot \frac{df}{dt}$ (i.e., gon4) performs the best, and the right subplot clarifies that this best performance is achieved using negative thresholds. This result suggests that, despite its poor individual performance, the insertion force can still be valuable for puncture detection when it is combined with other sensing modalities, such as reflectance.

#### 5.2.5 Ensemble component selection

By applying the selection criteria described in Supplementary Appendix 1.3, the 5512 signal-threshold combinations evaluated in the training set of insertions were pruned down to 56 optimal pairs and 56 base pairs for evaluation in the validation set. The 56 optimal pairs were ranked according to their DM, and the following three $P_{o}*$ were selected for application within the ensembles, as per Section 4.4.4:

1. $R_{1,u-\text{der}-h225}$ with λ=−283.7826 V/s (DM = 40.9),
2. $R_{5,s25-\text{der}-h175}$ with λ=3.0292 V/s (DM = 38.6), and
3. $f_{m9-\text{kow}-h175}$ with $\lambda =1.239\times 10^{-4}$ N (DM = 30.9).

For ease of reference, we henceforth refer to these three ensemble inputs as $e_{R1}$ , $e_{R5}$ , and $e_{f}$ , respectively.

### 5.3 Test set results

**FIGURE 9:**
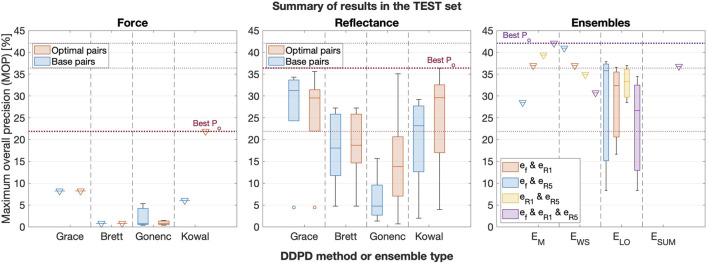
Box plots comparing the best force (left), reflectance (middle), and ensemble (right) performance in the test set, grouped by the DDPD method or ensemble type. Base pair results are included for each of the two sensors, and signal combinations are included for the ensembles. Triangular markers are added to boxes with zero standard deviation to improve their visibility.

The set of 56 base pairs, the set of 56 optimal pairs, and all ensemble permutations were applied to the test set of insertions. Figure 9 illustrates the performance of base and optimal pairs from each sensor in the test set (left and middle subplots for force and reflectance, respectively), and the right subplot shows ensemble results. Dotted horizontal lines emphasize the best performance from each group. Numerical results are provided in Table 4, which we report as our final results.

**TABLE 4:** Quantitative comparison of best optimal pairs and ensemble results in the test set of insertions.

Type	Signal	Ensemble identifier	$\lambda_{\text{MOP}}$	MOP (train) [%]	MOP (validate) [%]	MOP (test) [%]
Optimal pair Base pair	$f_{z,m9-\text{kow}-h175}$ $f_{z,m25-\text{der}}$	$e_{f}$ –	0.000 123 9 −4.9676	30.5 10.2	31.3 7.6	21.9 8.2
Optimal pair Base pair	$R_{4,s25-\text{kow}-h225}$ $R_{2,s47-\text{der}}$	– –	0.0155 10.1914	43.5 33.1	34.4 38.0	36.4 34.3
Optimal pair Optimal pair	$R_{1,u-\text{der}-h225}$ $R_{5,s25-\text{der}-h175}$	$e_{R1}$ $e_{R5}$	−283.7826 3.0292	41.2 37.6	40.6 39.6	35.6 29.5
Ensemble Ensemble Ensemble	E_M_ $\left(e_{f},e_{R1},e_{R5}\right)$ E_WS_ $\left(e_{f},e_{R5}\right)$ E_M_ $\left(e_{R1},e_{R5}\right)$	– – –	0.0245 16.506 764.6	41.1 40.2 42.9	51.0 48.4 37.5	42.1 41.0 39.4

## 6 Discussion

### 6.1 Major outcomes

The left subplot in Figure 6 illustrates that *all* DDPD approaches applied to reflectance can outperform *any* DDPD approach applied to force measured at the base of the needle, and Figure 9 confirms that the traditional force-only approach might not be very useful in the context of needle decompression and similar applications. Although force is the current gold standard for puncture detection, it tends to perform poorly within the context of DDPD for two reasons. First, tool-tissue interactions from non-target tissues can produce sensor features that overshadow the target puncture features. Second, variations in tissue properties and/or needle insertion velocities render the selection of generalizable static thresholds nearly impossible when relying on force alone. These two results, which have been previously observed by multiple DDPD researchers investigating other clinical applications, motivated our proposal to explore both reflectance and multimodal (ensemble) puncture-detection approaches.

Despite these issues, the performance of force-only DDPD schemes can be improved substantially with the inclusion of higher-frequency signal content, as suggested by Kowal (2017) and implemented here via HPFs. Table 4 confirms that the test set MOP increased from 8.2% for the best base pair in the traditional force-only approach to 21.9% in the best force optimal pair. Therefore, *a 2.7-fold performance improvement was achieved due to HPF application* in force data streams acquired from the base of the needle. In contrast, HPF’ing had a more modest effect in reflectance data streams: the test set MOP increased from 34.3% for the best reflectance base pair to 36.4% in its best optimal pair, i.e., only a 1.06-fold improvement.

Regardless, our results show that puncture detection using simple diffuse backscattered reflectance substantially outperforms any DDPD method applied to the output of a base-mounted force sensor–with or without the addition of an HPF. Indeed, the highest reflectance MOP from the test set was 4.4 times larger and 1.7 times larger, respectively, than the highest base pair MOP and the highest optimal pair MOP for force. Clearly, the inclusion of DRS provides substantial improvements over the standard approach.

Better yet, *multiplying data streams together (as proposed by Gonenc with position and force) yields even greater performance improvements*. Specifically, the best ensemble result provided a 5.1-fold improvement over the traditional force-only approach in the test set of insertions, which is an especially impressive result given the exceptionally lightweight nature of DDPD algorithms.

### 6.2 Limitations and future work

The baby back ribs used in this experiment had characteristics that are representative of human tissues, but they lacked the portion of the chest wall from the skin to the ribs. These missing tissues would have generated higher friction forces on the needle shaft, thus obscuring LoR sensations and potentially leading to higher overshoot. The tissue sample was stationary, it did not bleed or move, and it was ergonomically positioned for ease of insertion. These three factors likely decreased the difficulty of the needle insertions. The needle exit location was not clinically accurate given its exposure to ambient light, and it lacked the underlying tissues whose accidental puncture would have constituted an ND complication. To improve the clinical accuracy of future experiments, the needle must exit the tissue into a dark space filled with air, ideally with critical structures positioned just beyond the air gap. Such a configuration would allow for an evaluation of system utility in terms of the reduction of ND complications. Performance should also be evaluated during insertions that do not puncture the pleural membrane, which would test our proposed approach in the context of failed insertions.

Additionally, the laboratory environment employed in the reported study was far too quiet, calm, and comfortable to be representative of an emergency department or an accident scene. Increased realism during the ND task would almost certainly increase needle overshoot. Future experiments should attempt to heighten the realism by purposefully stressing and/or distracting operators. The robustness of the needle instrumentation should also be improved to protect the optical fibers from accidental breakage during more realistic use.

Though they are exciting, our results may not be representative of population means, since this validation experiment employed a single tissue sample and only two operators. Thus, our results are also limited by the small sample size. Although the quantitative results of this experiment yielded promising patterns, these patterns may not be reproducible in a more comprehensive data set. Furthermore, our results shed no light on the performance of expert operators. It will be important to test the clinical utility of puncture-detection notifications to identify scenarios where the instrumented needle-insertion adjunct we envision could be deployed to assist medical operators. As such, a large-scale user study is warranted; ideally, the study should include both naïve and expert participants to explore how reliably each type of operator can minimize overshoots based on tool sensations alone. It could also be beneficial to carefully compare the varied existing approaches employed by clinicians (e.g., syringe aspiration and/or auditory cues) with the DDPD methods proposed in this work.

## Data Availability

The raw data supporting the conclusions of this article will be made available by the authors, without undue reservation.
