# Supplementary material for: Enhancing needle puncture detection using high-pass filtering and diffuse reflectance
Source: Front Robot AI. 2025 May 6;12:1429327. doi: 10.3389/frobt.2025.1429327 (PMC12090360; doi:10.3389/frobt.2025.1429327)
Supplement: Supplementary file 1 [file DataSheet1.pdf]

Supplementary Material

1 APPENDIX

This appendix is for the article *Enhancing needle puncture detection using high-pass filtering and diffuse reflectance*, by R. L'Orsa, A. Bisht, L. Yu, K. Murari, G. R. Sutherland, D. T. Westwick, and K. J. Kuchenbecker.

1.1 Characterization of the DFDRS

To characterize the effects of ambient lighting on the DFDRS, we inserted the needle through an *ex vivo* sample of porcine intercostal tissue three times using a modulated light source. These three insertions were completed in a lab environment similar to that used for our main experiment, where the needle tip was exposed to open air after tissue puncture. We calculated the coefficient of variation (i.e., the ratio of the standard deviation to the mean: CV $= \frac{\sigma}{\mu}$) for 4 s-long data stream segments and averaged them across all three trials. The CV was 5.9×10^{-4}, which suggests that the DFDRS's output is not sensitive to the effects of ambient lighting.

An analytical sampling depth model proposed by Hennessy et al. (2014) was used to estimate the penetration depth (Z_S) of the light through each major layer of intercostal tissue. Their model is described mathematically as:

$$Z_S = a_1 + a_2 \left(\frac{1}{(1 + a_3 \cdot \mu_a)^{a_4}} \right) \left(\frac{1}{(1 + a_3 \cdot \mu_s')^{a_4}} \right), \tag{S1}$$

where μ_a and μ_s' are the absorption and reduced scattering coefficients of the tissue, respectively, and coefficients a_1 through a_4 are model-fitting parameters validated by Hennessy et al. (2014) from experimental data. Given the DFDRS's use of two 100 μm fibers with a source-detector separation (SDS) of about 125 μm, the relevant model parameters are $a_1 = 0.002325$, $a_2 = 0.032875$, $a_3 = 0.044891$, and $a_4 = 0.85$, as calculated using Hennessy et al.'s Table 2.

When a real ND is performed, the needle penetrates skin, fat, and muscle before puncturing the parietal pleura. However, we used porcine rib samples that already had the skin removed, meaning the needle only penetrated fat and muscle. As per Mosca et al. (2020), the absorption and reduced scattering coefficients of these tissues in response to 650 nm light are:

- Fat: μ_a = 0.70539 cm^{-1} and μ_s' = 6.34229 cm^{-1}
- Muscle: μ_a = 0.01304 cm^{-1} and μ_s' = 16.69901 cm^{-1}

Applying these values to Eq. (S1) yields sampling depths of 0.282 mm for muscle and 0.227 mm for fat, indicating that the DFDRS should achieve high spatial specificity in the main tissue types encountered during ND.

1.2 Criteria for removing insertions

During the frame-labeling process, twelve insertions were identified as inappropriate for DDPD algorithm application. Of these, five insertions did not produce pleural punctures, because either a) the operator stopped needle advancement too soon (one insertion), or b) the needle exited through a damaged portion of the tissue sample that lacked parietal pleura (four insertions). Four of the twelve outlier insertions involved

needle collisions with tissue-mount components, and the needle-exit location was obscured during three insertions (thus precluding frame labeling). Therefore, the full data analysis was applied to only 81 of the 93 needle insertions recorded in this study.

1.3 Criteria for advancement from the training set

Both threshold sets were applied to the axial insertion force and the five reflectance signal variations in the training set of insertions, and the P_o was identified for each signal variation. If multiple signal variations yielded the same value of MOP, the following procedure was used to select the set of P_o for advancement:

1. Find the set of P_o that does not include any HPF. If they all include an HPF, then keep them all.
2. In this set, keep all P_o from raw signals. If the set doesn't contain raw P_o, then keep all P_o with minimum l_{win} from the LPF.

These decision criteria help identify optimal pairs that might be better suited to real-time application because they include fewer filtering operations and/or smaller filter window sizes that add less signal-processing latency than other optimal pairs. Note that the final set of P_o^* for a given signal component could include, for example, the same LPF with multiple HPFs or two types of LPFs with the same window size. This selection process was also applied to the base pairs for comparison with the four prominent DDPD algorithms.

REFERENCES

Hennessy, R., Goth, W., Sharma, M., Markey, M. K., and Tunnell, J. W. (2014). Effect of probe geometry and optical properties on the sampling depth for diffuse reflectance spectroscopy. *Journal of Biomedical Optics* 19, 107002–107002

Mosca, S., Lanka, P., Stone, N., Konugolu Venkata Sekar, S., Matousek, P., Valentini, G., et al. (2020). Optical characterization of porcine tissues from various organs in the 650–1100 nm range using time-domain diffuse spectroscopy. *Biomedical Optics Express* 11, 1697–1706
